# Genetic and Molecular Evaluation: Reporting Three Novel Mutations and Creating Awareness of Pycnodysostosis Disease

**DOI:** 10.3390/genes12101552

**Published:** 2021-09-29

**Authors:** Khalda Sayed Amr, Hala T. El-Bassyouni, Sawsan Abdel Hady, Mostafa I. Mostafa, Mennat I. Mehrez, Domenico Coviello, Ghada Y. El-Kamah

**Affiliations:** 1 Molecular Genetics Department, National Research Centre, Cairo 12622, Egypt; khalda_nrc@yahoo.com; 2 Clinical Genetics Department, National Research Centre, Cairo 12622, Egypt; halabassyouni@yahoo.com; 3 Pediatric Department, Cairo University, Cairo 12613, Egypt; aragab80@gmail.com; 4 Oro-Dental Genetics Department, National Research Centre, Cairo 12622, Egypt; mostafanrc@yahoo.com; 5 Laboratorio di Genetica Umana, IRCCS Istituto Giannina Gaslini, 16147 Genova, Italy; domenicocoviello@gaslini.org

**Keywords:** cathepsin K, *CTSK* gene, dysmorphism, orodental, pancytopenia, pycnodysostosis

## Abstract

Pycnodysostosis is a rare autosomal recessive disorder with characteristic diagnostic manifestations. This study aims to phenotype and provide molecular characterization of Egyptian patients, with emphasis on identifying unusual phenotypes and raising awareness about pycnodysostosis with different presentations to avoid a mis- or under-diagnosis and consequent mismanagement. We report on 22 Egyptian pycnodysostosis patients, including 9 new participants, all descending from consanguineous families and their ages ranging from 6 to 15 years. In addition, prenatal diagnosis was performed in one family with affected siblings. They all presented with short stature, except for one patient who presented with pancytopenia as her primary complaint. Moreover, 41.2% of patients had sleep apnea, 14% presented with craniosynostosis, and 44.4% had failure of tooth development. Molecular analysis via direct exome sequencing of the cathepsin K gene revealed three novel mutations ((NM_000396.3) c.761_763delCCT, c.864_865delAA, and c.509G>T) as well as two previously reported mutations among nine new cases. The following is our conclusion: This study expands the molecular spectrum of pycnodysostosis by identifying three novel mutations and adds to the clinical and orodental aspects of the disease. The link between the *CTSK* gene mutations and the failure of tooth development has not been established, and further studies could help to improve our understanding of the molecular pathology.

## 1. Introduction

Pycnodysostosis is a rare osteosclerotic bone disease (MIM #265800) with an estimated prevalence of 1–3 per 1,000,000 [1]. It is caused by a homozygous mutation of the cathepsin K (*CTSK*) gene located on chromosome 1 and encodes for the lysosomal enzyme cathepsin K, a member of papain-like cysteine protease responsible for bone remodeling, whose deficiency leads to osteoclastic dysfunction [2]. Pycnodysostosis was first described by Montanari [3] and was then reintroduced by Maroteau and Lammy in 1962. In 1965, it was diagnosed as the cause of the disease of the Parisian artist Toulouse-Lautrec, with controversy resulting from the disease being named after him [4,5,6]. Pycnodysostosis follows an autosomal recessive pattern; hence, consanguinity is found in most reported families. However, the liability of chromosome 1 to undergo uniparental disomy, as another means of inheritance in pycnodysostosis, has been reported [7]. 

The main characteristics of radiographic disease are dense long bones, acro-osteolysis of the terminal phalanges and acral ends of the clavicles, open skull sutures, and straightening of the mandibular angle [8,9]. Pycnodysostosis could be confused with other forms of osteopetrosis with consequent inadequate management [1]. The crucial features of the disease include short-limbed short stature and stubby hands due to distal phalanges acro-osteolysis. Craniofacially, they show a hypoplastic maxilla and mandible, as well as frontal and bi-parietal bossing, proptotic eyes, and delayed closure of cranial sutures. Orodental manifestations include palatal grooving, dental crowding, and hypodontia. Early eruption of deciduous teeth and delayed eruption of their successors have been also reported [6]. Intelligence is characteristically normal with mild psychomotor problems described in some individuals [10].

Several unusual features of pycnodysostosis have been reported: craniosynostosis, sleep apnea, pectus carinatum, osteosarcoma, ichthyosis vulgaris, obesity, hearing loss, and multiple pituitary hormone deficiencies [7,11,12,13,14,15,16,17].

The low prevalence of pycnodysostosis, combined with its molecular and clinical heterogeneity, makes it difficult to define a classical phenotype and to recommend proper patient monitoring and management decisions. Here, we describe the clinical and molecular findings of 22 Egyptian patients including 9 newly diagnosed patients, describing variable phenotypes associated with germline *CTSK* variants.

## 2. Patients and Methods

Twenty-two Egyptian patients (thirteen previously reported) [referenced in Table 1] are compared phenotypically, in correlation to their underlying molecular pathologies. Among them are nine new patients (five females and four males) and one amniotic fluid (AF) sample from a pregnant mother of previous pycnodysostosis siblings, all descending from consanguineous families clinically diagnosed with pycnodysostosis, who were recruited from the Hereditary Blood Disorders (HBD), the Clinical Genetics and the Oro-dental Genetics clinics, and the National Research Centre, Cairo, Egypt. Patients were included based on the presence of the cardinal clinical and radiological disease manifestations. Detailed medical history was recorded. Three-generation pedigree analyses were performed, and an assessment of demographic data, history of present illness, and disease progression, in addition to a thorough clinical evaluation including anthropometric measurements and dermatological and orodental examination, was performed. Five-milliliter peripheral blood samples were collected in EDTA tubes from all recruited patients and their available family members for genomic DNA extraction. Samples were collected after obtaining informed written consents according to the Helsinki Declaration of 1975, as revised in 1983, and the Institutional Review Board, National Research Centre.

DNA was extracted using a QIA-gene extraction kit, according to the manufacturer’s protocol. QIAmp DNA blood mini kit (Qiagen Inc., Valencia, CA, USA) was performed, and polymerase chain reactions (PCRs) were used to amplify *CTSK* gene exons and its flanking sequences. Primer sequences of *CTSK* gene exons are listed in Table 2.

PCR products were purified using a QIAquick PCR purification kit (Qiagen, Redwood City, Germany). Forward and reverse DNA strands were sequenced using the BigDye Termination kit (Applied Biosystems, Foster City, CA, USA) and analyzed on the ABI Prism 3500 Genetic Analyzer (Applied Biosystems) according to manufacturers’ protocols. The attained nucleotide sequences were aligned and compared with a reference gene (NM_000396.3). Variants were named according to Human Genome Variation Society (http://www.hgvs.org, accessed on 30 July 2021). The pathogenicity of the new variants on protein function was checked through in silico analysis using different bioinformatics tools (Table 3). The novel gene variants were co/nfirmed after ruling out the polymorphisms through concurrent analysis of 100 Egyptian healthy individuals.

## 3. Results

### 3.1. Phenotypic Results

**Figure 1:**
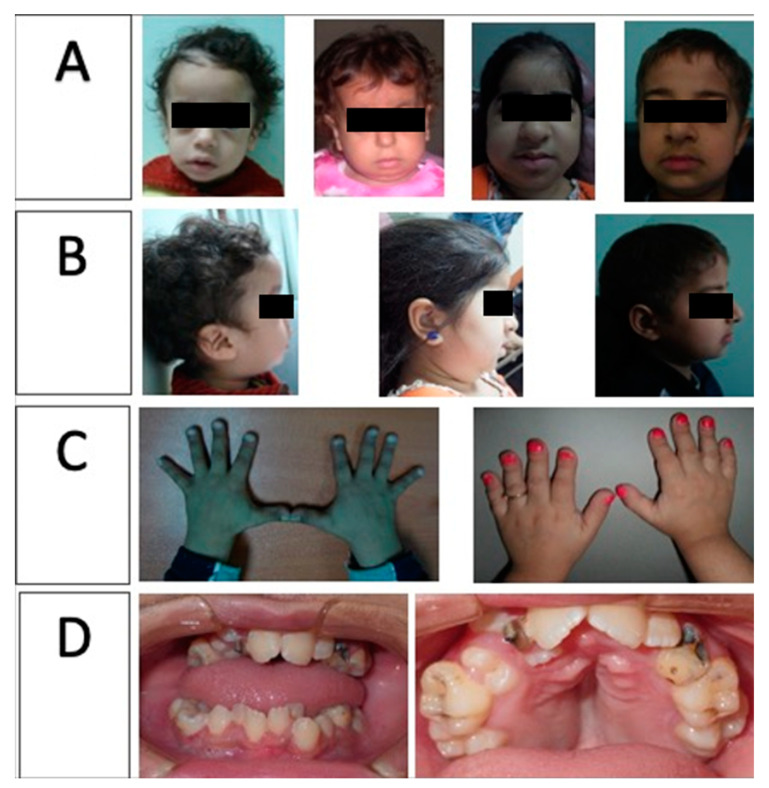
(**A**) Frontal view: characteristic facies; (**B**) profile view: beaked nose and hanging columellas; (**C**) stubby hands with bulbous and dystrophic nails; (**D**) crowding of teeth, malposition, and collapsed palatal shelves with midline grooving.

The nine new cases all presented with a characteristic pycnodysostosis phenotype: short stature and characteristic facies (Figure 1). Some patients presented with unusual phenotypes: patient 7 presented with pancytopenia and bone marrow depression; patient 6 had crowded teeth (Figure 1D); patient 4 complained mainly of sleep apnea, which was also reported in patients 19, 20, 21, and 22; patients 5, 7, 9, and 14 presented with craniosynostosis. Only one patient (patient 5) among the new 9 cases had a femur fracture following a fall down the stairs. Five of the previously reported cases had a history of fracture (once: patients 12, 19, and 22; three times: patient 13; and nine times: patient 17). All of the patients’ data are listed in Table 1.

### 3.2. Radiologic Results

**Figure 2:**
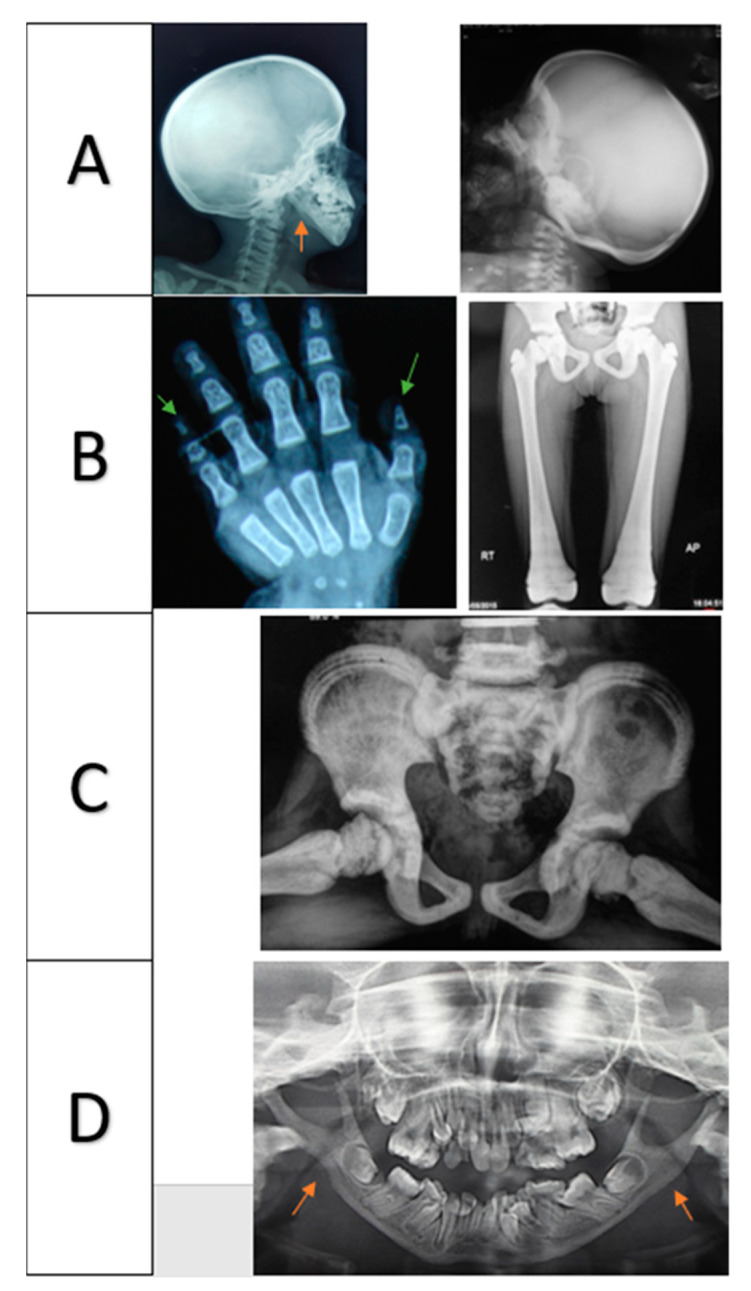
(**A**) Skull showing thick calvarium, open anterior fontanelle, and wormian bones; red arrows indicate straight mandibular angles. (**B**,**C**) Increased bone density; green arrows indicate acro-osteolysis of terminal phalanges. (**D**) Panorama X-rays showing crowding of the teeth; red arrows indicate straight mandibular angles.

Characteristic radiological findings of pycnodysostosis were present in all 22 cases, including increased density of long bones, acro-osteolysis of terminal phalanges, and straight mandibular angle (Figure 2A–D). Open anterior fontanelle was only present in 76% of cases. The anterior fontanelle was prematurely closed in cases 5 (6 m), 11 (7 m), 12 (9 m), and 14 (7 m). Dental crowding and hypodontia were revealed in the panorama X-rays (Figure 2D).

### 3.3. Molecular Results

**Figure 3:**
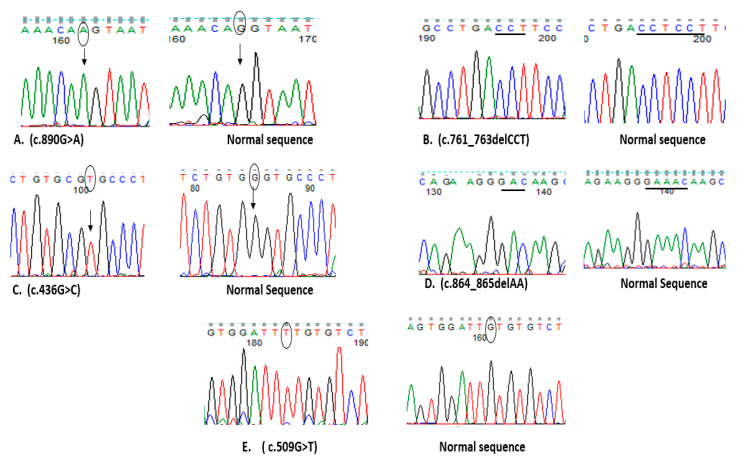
Direct sequencing of *CTSK* gene obtained from nine patients compared to those from an unrelated normal control. (**A**) c.890G>A was found in patients 1 and 2. (**B**) 3-bp(CCT) deletion at nt 761 was found in patients 3, 4, 5, and 6. (**C**) c.436G>C was found in patient 7. (**D**) 2-bp(AA) deletion at nt 864 was found in patient 8. (**E**) c.509G>T was found in patient 9.

**Figure 4:**
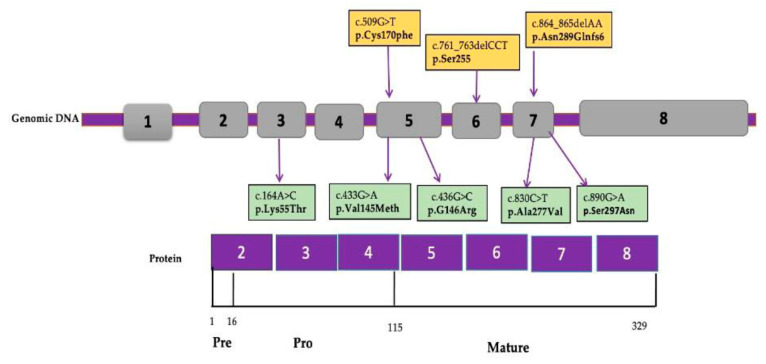
*CTSK* exons and their corresponding protein domains. *CTSK* gene has 8 exons encoding 329 amino acids, and numbers refer to amino acid positions. Eight mutations were identified: three novel mutations (yellow boxes) and five previously reported mutations (green boxes).

Five variants including three described for the first time, namely, c.509G>T (p.Cys170Phe), c.761_763delCCT (p.Ser255), and c.864_865delAA (p.Asn289Glnfs*6) (Table 2 and Table 3), were characterized among the nine new participants. Accordingly, a total of eight different variants were found among all reported molecularly studied (19/22) patients (Table 4 and Figure 3 and Figure 4).

The three new mutations were not found in the dbSNP, 1000G, ExAC, and genome-AD. They were deemed pathogenic by various bioinformatic tools (Mutation Taster, Sift, Polyphen2) and could not be detected among the studied 100 chromosomes of healthy Egyptian controls, confirming the notion that they are not polymorphisms within the Egyptian population. Moreover, the amniotic sample in one family member revealed a biallelic-affected causative variant similar to his affected sibling; the mother was accordingly counselled with termination of pregnancy.

### 3.4. Protein Function Prediction Analysis of c.509G>T

**Figure 5:**
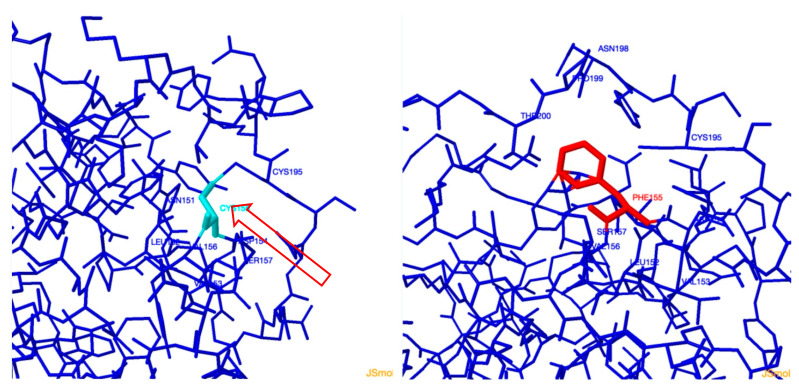
Three-dimensional structure analysis using missense 3D showing the effect of the new missense mutation c.509G>T (p.Cys170phe) in CTSK gene causing pycnodysostosis. Red arrow referring to the disulphide bond in the wild type protein structure which will get lost in the mutant protein. The cyan structure is representing the Cysteine residue in the wildtype protein and the red structure is representing the phenylalanine residue in the mutant structure.

Three-dimensional structure analysis using the missense 3D online-available tool (Figure 5) showed that the p.Cys170phe substitution triggers a clash alert. The local clash score for the wild type is 19.58, and the local clash score for the mutant is 38.83, which will reduce the molecule mobility of the protein. Furthermore, this substitution leads to the loss of the disulfide bond between 170 and 210, which is naturally occurring in the wildtype of this protein, and consequently results in a reduction in the protein stability as well as altering the cathepsin K domain function.

## 4. Discussion

The current study presents the largest cohort of pycnodysostosis in Egyptian patients, thoroughly discussing variable presentations, complications, and molecular pathology with a report of three novel CTSK disease-causing variants. Our report describes 22 Egyptian pycnodysostosis patients with female preponderance (60%) whose ages ranged from 4 to 39 months at disease onset and 6–15 years at reporting time. All participants were descendants from consanguineous families, which is in accordance with the autosomal recessive nature of the disorder [1]. Considering the number of patients collectively reported in Egypt, the incidence would be about 1/4–4.5,000,000, which is less than international reports even in countries with low incidence of consanguineous marriages, despite the high consanguinity rates among Egyptians (55.9%) [22,23].

This relatively small cohort of Egyptian patients might be explained by misdiagnosis, due to overlapping with other osteopetrosis patients, or due to unusual features in some pycnodysostosis patients [1,7,11,12,13,14,15,16,17]. Among those unusual presentations detected in our studied cohort are pancytopenia and bone marrow failure, which were the complaints of patient 7 presenting at the Hereditary Blood Disorders clinic. Reviewing the literature, pycnodysostosis patients usually suffer no change in their blood work-up, which is one of the factors that differentiates it from osteopetrosis, both being disorders of osteosclerosis [18]. Similarly, in a study of 27 benign osteopetrosis patients, whole-exome sequencing diagnosed six pycnodysostosis patients, which was the first documentation of pycnodysostosis patients suffering from pancytopenia [24]. We herein confirm the liability of pancytopenia occurring in pycnodysostosis patients.

The absence of the mandibular angle, a feature that is highly diagnostic and differentiates pycnodysostosis from cleidocranial dysplasia, was present in all our reported patients (Figure 1B). However, adding to the phenotypic heterogeneity, previous investigators asserted that not all of their studied patients showed the absent mandibular angle, which is present in 84–94% of published cases [6,9].

Another infrequent presentation was sleep apnea as a complaint. Sleep apnea was not unusual among our studied cohort, being present in 5/13 (41.7%) of the cases who were questioned about sleep apnea: one of the nine new participants (patient 4) and in patients 19, 20, 21, and 22. The wide age range of diagnosis of pycnodysostosis, which in some cases reaches up to 50 years, makes the reasons prompting patients to seek help rather intriguing. Pycnodysostosis patients usually seek medical help because of short stature or unusual bone fracture [25]. In some families, short stature can remain unnoticed or treated as a normal condition for years until a more pressing condition presents itself, whether as an atypical fracture or sleep apnea [26].

Short stature is a cardinal phenotype of pycnodysostosis, and some studies have reported reduced growth in pycnodysostosis patients possibly due to lower levels of IGF-1 [8]. However, low levels of GH and IGF-1 were only detected in one of the newly added participants (patient 8), who reached a height close to normal for his age after treatment with GH therapy.

Hypodontia is regarded as a secondary feature in pycnodysostosis syndrome, as it is not usually present in all cases. This also discourages a diagnosis of cleidocranial dysplasia, which is a disorder of supernumerary teeth. Hypodontia was detected in four out of the nine newly studied patients. The link between *CTSK* gene mutations and the failure of tooth development has not yet been established, and further studies are warranted in this area to help in furthering our understanding of the molecular pathology of the disease. In contrast, the palatal affection, particularly the collapsed palatal shelves with midline grooving, is a constant feature of this disorder (Figure 1D) [9,27].

It was reported that mutations of the *CTSK* gene displayed multiple dental abnormalities, such as hypoplasia of the enamel, obliterated pulp chambers, and periodontal disease, none of which were found among our studied patients [28]. The observed dental findings included a high caries index of 75%, which could be attributed to the crowding and malposition of teeth. Open bite was present in 71.4% of cases, which is another consequence of the crowding and malposition of teeth. It is worth noting that dental crowding was one of the patient’s chief complaints. This elucidates the importance of orodental examination in patients with pycnodysostosis syndrome.

Characteristic radiologic findings, including dense long bones, acro-osteolysis of the terminal phalanges and acral ends of the clavicles, open skull sutures, and straightening of the mandibular angle, were detected in variable percentages among our discussed cases [8,9]. Dense bones were present in all of our studied cases; it was suggested the pycnodysostosis patients’ bones are stronger than the reference values from the general population. However, several studies reported recurrent fractures among pycnodysostosis patients, including our study, where patients 3, 5, 12, 19, and 22 were fractured once. Patients 13 and 17, who suffered recurrent fractures (3 and 9, respectively), both harbored different mutations, namely, c.436G>C and c.164A>C respectively; however, patients 12 and 16 did not have any history of recurrent fracture despite harboring the same mutation as patient 17, which suggests the absence of genotype/phenotype correlation. However, Otaify et al. 2018 recorded a high incidence of pathological fractures contrary to the reports of Doherty et al. [18].

The increase in bone density created significant scientific interest in targeting the enzymatic activity of the *CTSK* gene to increase bone density in patients with osteoporosis [23,29,30,31,32,33]. Bone et al. [32] and Pirapaharan et al. [34] concluded in their studies that low doses of cathepsin K inhibitors increase the levels of CTSK; however, higher doses decrease these levels.

Craniosynostosis was another unusual phenotype detected in three of our discussed cohort (patients 5, 11, and 14). Open fontanels, a usual finding in pycnodysostosis, were detected in 76.2% of our patients, which corroborates previous findings [6,35,36]. However, Song et al. [26], described a 41-year-old female with a closed anterior fontanel, mandibular angles that appeared normal, and fingers that were not stubby; this is where a molecular study is the sole diagnostic tool to exclude other osteopetrosis syndromes. Bulbous broad nails were present in 71.4% of the current study participants (Figure 1C).

Our molecular results shed light on the genetic alterations within the cathepsin K gene in the nine studied participants and the distribution of variants observed in all 19 patients with underling genetic testing (our cohort plus previously reported Egyptian cases) (Figure 3 and Figure 4). The substitution of both glycine and serine residues at amino acid positions G146 and 297 in patients 1, 2, 7, 10, 11, 13, 14, and 18 was observed in variants in our cohort as well as in previously reported Egyptian patients, with a total of 8/19 (42.1%) patients carrying either the common p.Gly146Arg or the rare p.Ser297Asn in reported mutations of the *CTSK* gene. The first mutation, c.436G>C (p.Gly146Arg) characterized in 3/19 (15.8%) of the Egyptian patients and affecting the residue of the mature domain in *CTSK* gene, yielded a mature form of a nonfunctional protein and had been previously found in many pycnodysostosis families from diverse ethnic backgrounds: Moroccan Arab, American Hispanic, Tunisian, Algerian, and Egyptian patients [18,19,21,37,38]. The second rare reported point mutation, c. 890G>A (p.Ser297Asn) or r.785_890del causing skipping of the entire exon 7, was found in 5/19 (26.3%) of the Egyptian patients, supporting the hypotheses that this splice mutation is specific to Egyptians, and the association of this particular recurrent Egyptian mutation for different period of time supports the pan-ethnic nature of the disease.

Similarly, recurrent pathogenic variants in different ethnic populations have been reported with a suggestion of a founder effect as described in Denmark, Brazil, and Turkey [2,39,40].

The unreported newly detected deleterious mutation characterized in patient 9, c.509G>T(p.Cys170Phe), was located in exon 5,which was predicted to be highly conserved along species and resulted in altered protein product (Figure 5). Cys170 codon was previously reported with a different change to serine [41].

Interestingly, the novel in-frame deletion mutation c.761_763delCCT was found in patients 3, 4, 5 and 6, and it is worth noting that patient 3 had a different clinical finding of pancytopenia, which suggests that either there is no genotype phenotype correlation or she has another modifier variant causing an impact on the appearance of such phenotype.

A novel frameshift mutation, c.864_865delAA, was detected in patient 8; the two new deletions are located in the mature active domain of the protein [42]. According to the Human Mutation Database, only four small deletion mutations were reported in the *CTSK* gene in different ethnic backgrounds: c.259delG, c.266-268delAGA, c.426delT, and c.737_738delCT [24,43,44]. The distribution of the identified mutations in the *CTSK* gene database showed that around 70% of the mutations were found in the mature domain, and all identified mutations in the current study are located within the last 215-amino acid mature domain (7/8, 87.5%,) with a first report of a frameshift deletion in this important domain containing the catalytic active site of the CTSK protein. In our previous report [18], only one mutation, p.Lys55Thr (1/8, 12.5%) was detected in three patients in the proregion of the CTSK protein that interacts with the substrate binding groove and along the proregion binding loop (residues Ser138–Asn156) (Figure 5).

Expanding the molecular spectrum through the three newly detected pathogenic mutations postulates an additional verification of the molecular heterogeneity underlying this skeletal dysplasia worldwide and, specifically, in Egypt, since the appearance of recurrent mutations among Egyptian families disrupts this hypothesis.

Molecular results helped provide counseling and prenatal analysis of the targeted causative variant in family 2, where an affected homozygous was diagnosed prenatally, and the mother decided to terminate the pregnancy.

## 5. Conclusions

The current study is a thorough genetic report of Egyptian pycnodysostosis patients that expands the molecular spectrum by reporting three novel mutations. Pycnodysostosis is usually diagnosed due to its remarkable skeletal and facial dysmorphism, but our study elucidated the unusual phenotypes to be considered so as to not mis- or under-diagnose the disorder. Furthermore, in certain instances, diagnostic key features may take late ranking, such as as short stature, which could pass with no concern in some families. Thorough clinical, orodental, and radiological examination is important, as well as molecular analysis, for verification and proper patient counselling.

Although pycnodysostosis has been thoroughly investigated orodentally, there are many disorders that remain unexplored from this perspective. Orodental geneticists could help improve phenotyping and ultimate diagnosis.

## Figures and Tables

**Table 1:** Clinical, Radiological, Biochemical, Oro-dental and Molecular data for all 22 Patients.

	1	2	3	4	5	6	7	8	9	10	11	12	13	14	15	16	17	18	19	20	21	22
Age in months	9	15	9	7	6	10	6	8	6	7	9	17	39	7	6	4		NA	5	7	4	5
Sex	F	M	F	M	F	F	F	M	M	F	F	F	F	F	M	F	M	M	F	M	M	F
Consanguinity	+	+	+	+	+	+	+	+	+	+	+	+	+	+	+	+	+	+	+	+	+	+
Short stature	+	+	+	+	+	+	+	+	+	+	+	+	+	+	+	+	+	NA	+	+	+	+
Frontal Bossing	+	+	+	+	+	+	+	+	+	+	+	+	+	+	+	+	+	NA	+	+	+	+
Open Fontanelle	+	+	+	+	-ve	+	+	+	+	+	-ve	-ve	-ve	-ve	+	+	+	NA	+	+	+	+
Brachydactyly	+	+	+	+	+	+	-ve	+	+	+	+	+	+	+	+	+	+	NA	+	+	+	NA
Micrognathia	+	+	+	+	-	+	+	+	-ve	+	+	+	+	+	+	+	+	NA	+	+	+	NA
Prominent nose	+	+	+	+	-	+	-ve	+	-ve	+	+	+	+	+	+	+	+	NA	+	+	+	NA
Dystrophic Flat Nails	+	+	+	+	-	+	+	-ve	-ve	+	+	+	+	+	+	+	+	NA	+	+	+	NA
Straight mandibular angle	+	+	+	+	+	+	+	+	+	+	+	+	+	+	+	+	+	NA	+	+	+	NA
Dental Caries	+	-ve	+	+	+	+	+	+	+	-ve	+	-ve	-ve	-ve	-ve	-ve	+	NA	+	+	+	+
Open Bite	+	-ve	+	+	+	+	-ve	+	+	+	+	+	+	+	+	+	+	NA	NA	NA	NA	NA
Hypodontia	+	-ve	-ve	-ve	+	+	-ve	+	+	+	+	-ve	-ve	-ve	-ve	+	-ve	NA	+	+	+	+
Previous Fractures	-	-ve	-ve	-ve	+	-ve	-ve	-ve	-ve	-ve	-ve	+ (1)	(3X)	-ve	-ve	-ve	(9X)	NA	+ (1)	-ve	-ve	+
Pancytopenia /BM depression	+	-ve	-ve	-ve	-	-ve	+	-ve	-ve	NA	NA	NA	NA	NA	NA	NA	NA	NA	-ve	-ve	-ve	NA
Sleep Apnea		-ve	-ve	+		-ve	-ve	-ve	-ve	NA	NA	NA	NA	NA	NA	NA	NA	NA	+	+	+	+
NO. of exon	7	7	7	6	6	6	6	7	5	7	7	3	5	5	5	3	3	7	NA	NA	NA	7
Location of CTSK cDNA	c.890G>A	c.890G>A	c.761_763delCCT	c.761_763delCCT	c.761_763delcct	c.761_763delCCT	c.436G>C	c.864_865delAA	c.509G>T	c.890G>A	c.890G>A	c.164A>C	c.436G>C	c.436G>C	c.433G>A	c.164A>C	c.164A>C	c.890G>A	NA	NA	NA	c.830C>T c.830C>T
Amino Acid Change	p.Ser297Asn	p.Ser297Asn	p.Ser255	p.Ser255	p.Ser255	p.Ser255	p.Gly146Arg	p.Asn289Glnfs6	p.Cys170phe	p.5er297Asn	p.5er297Asn	p.Lys55Thr	p.Gly146R	p.G146Arg	p.Val145Meth	P.Lys55Thr	p.Lys55Thr	P.Ser297Asn	NA	NA	NA	p.Ala277Val
Mutation type	Missense	Missense	Inframe deletion	Inframe deletion	Inframe deletion	Inframe deletion	Missense	Frameshif	Missense	Missense	Missense	Missense	Missense	Missense	Missense	Missense	Missense	Missense	NA	NA	NA	Missense

Patients (1–9) the current study, Patients (10–17) by Otaify et al., 2018 [18], (Patients (18) by Donnarumma et al., 2007 [19], Patients (19–21) by Abdallah et al., 2012 [20], Patient (22) by Bizaoui et al., 2019 [1]. Male (M); Female (F); Not available (NA); Positive (+); Negative (-ve); (+3) & (+5) number of fractures.

**Table 2:** Primer sequences of *CTSK* gene exons.

	F	R
Exon 2	CCAGCATCCTATCTAAACACAGG	GTCTCAGCCTTCCTGCCATG
Exon 3	GATTGTGAGTTTCCTTTATTCTCC	GCATCAGCAGGGAACTAAAG
Exon 4	GCTTTAGTTCCCTGCTGATGC	GGAAAAGGTCATGCCAGATTAC
Exon 5	CACATGGAATTTCTTCAGGC	CATCATGCTGGGGAAGGAG
Exon 6	GCTGCCTCTGTTAGTTCACTG	GACAGTGCTGTATAGGATCAGC
Exon 7	GCTGATCCTATACAGCACTGTC	GAAAGGAATATCGGGAAGCTG
Exon 8	GTGTACCATCAGTACCTCGCAC	CTCAGTATCACCACATCTGCTTC

**Table 3:** The characteristics of the new *CTSK* gene mutations in the studied patients.

cDNA Change	Protein Change	Mutation on Type	SIFT	Polyphen2	Mutation Taster	ACMG-AMP	PhD-SNP	Mutation Assessor	PROVEAN	SNPs&GO	REVEL	MutPred
c.509G>T	p.Cys170phe	Missense	Damaging (0)	Probably Damaging (1)	Disease causing (1)	3(PM2, PP3)	Disease (0.936, RI = 9)	High (4.69)	Deleterious (−10.223)	Disease (0.873, RI = 7)	Damaging effect (0.973)	0.88 Damaging (0.887) Gain of catalytic residue at C170 (*p* = 0.0112)
c.761_763 delcct	p.Ser255	Inframe deletion	NA	NA	Disease Ccusing	5(PVS1, PM2, PP3	NA	NA	NA	NA	NA	NA
c.864_865delAA	p.Asn289Glnfs6	Deletion	NA	NA	Disease causing	5(PVS1, PM2, PP3	NA	NA	NA	NA	NA	NA

N/A = not applicable; PM2 = Pathogenic Moderate 2; PP3 = Pathogenic Supporting 3; PVS1 = Pathogenic Very Strong 1; SIFT = Sorting Intolerant from Tolerant. PolyPhen2 score ranges from 0.0 (tolerated) to 1.0 (deleterious). Mutation Taster: disease causing or polymorphism. Abbreviations: Mutation Assessor, predicted functional, i.e., high (“H”) or medium (“M”), or predicted non-functional, i.e., low (“L”) or neutral (“N”). Score cutoffs between “H” and “M”, “M” and “L”, and “L” and “N” are 3.5, 1.935 and 0.8, respectively; NE, not evaluated; PhD-SNP and SNPs&GO directly predict disease effect and give reliability index (RI) range from 0 to 10; 0 = unreliable and 10 = reliable. PROVEAN score ≤ −2.5 is predicted as “Damaging”; otherwise, it is predicted as “Neutral”. REVEL, PolyPhen2, and MutPred score ranges from 0.0 (tolerated) to 1.0 (deleterious); predicted effect on the molecular mechanisms with *p* ≤ 0.05 (probability; *p*-value) also given. SIFT threshold for intolerance is 0.05. According to ACGM-AMP guidelines of variant interpretation, this variant causes an unambiguous pathogenic effect on the CTSK protein (frame shifting) c.864_865delAA p.Asn289Glnfs6.

**Table 4:** The characteristics of *CTSK* gene mutations in this study.

Patient	Exon	Domain	cDNA Change	Protein Change	Mutation Type	References
1	7	Mature domain	c.890G>A	p.Ser297Asn	Missense	Donnarumma et al. 2007 [19]
2	7	Mature domain	c.890G>A	p.Ser297Asn	Missense	Donnarumma et al. 2007 [19]
3	6	Mature domain	c.761_763delCCT	p.Ser255	In-frame deletion	This study
4	6	Mature domain	c.761_763delCCT	p.Ser255	In-frame deletion	This study
5	6	Mature domain	c.761_763delCCT	p.Ser255	In-frame deletion	This study
6	6	Mature domain	c.761_763delCCT	p.Ser255	In-frame deletion	This study
7	5	Mature domain	c.436G>C	p.Gly146Arg	Missense	Gelb et al. 1998 [21]
8	7	Mature domain	c.864_865delAA	p.Asn289Glnfs6	Frameshift	This study
9	6	Mature domain	c.509G>T	p.Cys170phe	Missense	This study

## Data Availability

All patients’ data are included in the manuscript.
